# Promoting Resilience to Food Commercials Decreases Susceptibility to Unhealthy Food Decision-Making

**DOI:** 10.3389/fpsyg.2020.599663

**Published:** 2020-12-02

**Authors:** Oh-Ryeong Ha, Haley J. Killian, Ann M. Davis, Seung-Lark Lim, Jared M. Bruce, Jarrod J. Sotos, Samuel C. Nelson, Amanda S. Bruce

**Affiliations:** ^1^Department of Psychology, University of Missouri–Kansas City, Kansas City, MO, United States; ^2^Department of Pediatrics, University of Kansas Medical Center, Kansas City, KS, United States; ^3^Center for Children's Healthy Lifestyles & Nutrition, Kansas City, MO, United States; ^4^Department of Biomedical and Health Informatics, University of Missouri–Kansas City, Kansas City, MO, United States

**Keywords:** food decisions, eating behavior, advertising literacy, children, obesity, food commercials

## Abstract

Children are vulnerable to adverse effects of food advertising. Food commercials are known to increase hedonic, taste-oriented, and unhealthy food decisions. The current study examined how promoting resilience to food commercials impacted susceptibility to unhealthy food decision-making in children. To promote resilience to food commercials, we utilized the food advertising literacy intervention intended to enhance cognitive skepticism and critical thinking, and decrease positive attitudes toward commercials. Thirty-six children aged 8–12 years were randomly assigned to the food advertising literacy intervention or the control condition. Eighteen children received four brief intervention sessions via video over 1 week period. In each session, children watched six food commercials with interspersed embedded intervention narratives. While watching food commercials and narratives, children were encouraged to speak their thoughts out loud spontaneously (“think-aloud”), which provided children's attitudes toward commercials. Eighteen children in the control condition had four control sessions over 1 week, and watched the same food commercials without intervention narratives while thinking aloud. The first and last sessions were held in the laboratory, and the second and third sessions were held at the children's homes. Susceptibility to unhealthy food decision-making was indicated by the decision weights of taste attributes, taste perception, food choices, *ad libitum* snacking, and cognitive and affective attitudes toward food commercials. As hypothesized, the intervention successfully decreased susceptibility to unhealthy food decision-making evidenced by reduced decision weights of the taste in food decisions, decreased tasty perception of unhealthy foods, and increased cognitive skepticism and critical thinking toward food commercials. In addition, as children's opinions assimilated to intervention narratives, their cognitive skepticism and critical thinking toward commercials increased. The aforementioned results were not shown in the control condition. However, this brief intervention was not enough to change actual food choices or food consumption. Results of this study suggest that promoting resilience to food commercials by enhancing cognitive skepticism and critical thinking effectively reduced children's susceptibility to unhealthy food-decision making.

## Introduction

Children are highly susceptible to unhealthy foods. Pre-disposed sweet and salty taste preferences and bitter and sour taste rejections, innate preferences for high caloric foods, and early experience rewarding those predispositions make children be inclined to eat unhealthy foods high in sugar, salt, and fat (Birch and Fisher, 1998; Mela, 2001; Beauchamp and Mennella, 2009; De Cosmi et al., 2017). As previous food decision research has shown, children primarily incorporate taste attributes, while they barely consider health attributes (Bruce et al., 2016; Lim et al., 2016; Ha et al., 2019). Such heavily weighted taste-oriented food decisions are often linked to unhealthy food preferences, overeating, and a risk of developing obesity in children and adolescents (Neumark-Sztainer et al., 1999; Shannon et al., 2002; Boyland and Halford, 2013).

Food commercials add more layers of complexity to healthy eating, and children are vulnerable to the undesired effects of advertising. Exposure to food commercials provokes hedonic food cue processing and eating behavior on multiple levels including heightened visual attention to unhealthy foods (Spielvogel et al., 2018), hedonic eating (Harris et al., 2009), requests for and consumption of the advertised foods (Gorn and Goldberg, 1982; Utter et al., 2006), and preference for and consumption of high-fat, high-sugar, energy-dense foods (Boyland et al., 2011, 2016). Children-targeted advertising featuring high-caloric, low-nutrient food are related to the prevalence of childhood obesity (Linn and Novosat, 2008; Goris et al., 2010). Even exposure to commercials featuring healthier meal options of familiar fast food brands or commercials featuring unfamiliar fast foods with healthy messages failed to improve food healthiness perception but resulted in increased fast food preferences (Boyland et al., 2015; Harris et al., 2018). Neuroimaging research has demonstrated that food brand logos have high attentional salience (Masterson et al., 2019b), and food brand logos and food commercials activate the brain's reward system (Bruce et al., 2014, 2016; Gearhardt et al., 2020; Ha et al., 2020). The greater activation of the reward system often links to overeating and body fat gain in children and adolescents (Stice and Yokum, 2016; Adise et al., 2018).

Enhancing resilience to the adverse effects of food commercials could be critical for the development of healthy eating habits and weight management in children and adolescents. While limiting food commercials and media time would reduce the chances to be exposed to harmful advertising effects (Smith et al., 2019), establishing life-long strategies for regulating eating decisions in the presence of unhealthy food cues in commercials during the course of development could increase resilience to food advertising (Buijzen and Valkenburg, 2005). Advertising literacy is one of the abilities central to children's understanding of marketing (Malmelin, 2010). The response and understanding of advertising includes cognitive and affective components (Burton and Lichtenstein, 1988). Advertising literacy consists of cognitive advertising literacy, for increasing understanding selling, persuasive intent and advertising skepticism, and affective advertising literacy, for increasing negative affective attitudes toward commercials (Rozendaal et al., 2011; Hudders et al., 2016). Children develop a rudimentary understanding of advertisements as a differentiated entity after 5 years of age, and their understanding of selling and persuasive intent and tactics develops between 8 and 12 years of age (Blosser and Roberts, 1985; Livingstone and Helsper, 2006). Children's understanding of advertising literacy is poor until adolescence (Oates et al., 2002; Rozendaal et al., 2010) and develops at a pace consistent with other cognitive and information processing capacities (Moses and Baldwin, 2005; Hudders et al., 2016). The activation of advertising literacy knowledge as a cognitive defense is not spontaneous and requires retrieval cues for 8- to 12-years-old children (Brucks et al., 1988; Rozendaal et al., 2012). Children in this age range (8–12 years) are most affected by televised food marketing (Gantz et al., 2007). Intervention strategies using advertising literacy narratives or information as cues to activate advertising literacy have been shown to effectively enhance defenses against adverse advertising effects in children (Buijzen, 2007; Rozendaal et al., 2016; De Jans et al., 2017). Particularly, factual (cognitive) narratives are shown to increase cognitive defenses by delivering advertising knowledge and skepticism. Increased advertising knowledge and skepticism decrease susceptibility to commercials (i.e., attitude toward the brand and products, such as intended product request) by increasing negative attitudes toward the commercials. Evaluative (affective) narratives decrease susceptibility to commercials by increasing negative attitudes and facilitating negative affective responses (Buijzen, 2007). When children critically process advertising using “think-aloud” approaches, which encourage spontaneous speech, they exhibit both increased cognitive defenses and negative affective attitudes that decrease their susceptibility to commercials (Rozendaal et al., 2012).

Research has mainly examined the effect of advertising literacy interventions in decreasing positive attitudes toward the advertising and susceptibility to the commercials from the perspective of consumer behaviors. Considering that exposure to television food commercials increases food consumption (Harris et al., 2009; Russell et al., 2019) and contributes to the development of childhood obesity (Kelly et al., 2010), it is important to test how advertising literacy interventions influence food decision-making and consumption to prevent obesity. Specifically, promoting resistance to advertising effects on food taste attributes will ultimately be beneficial for healthy eating. Children show strong taste preferences to advertised foods. Children perceive that the same foods taste better when those foods are in fast-food brand or cartoon character packaging, especially when children have more frequent television exposure and fast food consumption experiences (Robinson et al., 2007; Enax et al., 2015). Furthermore, our previous research has shown that exposure to food commercials increases the relative importance (decision weights) of taste attributes in food decisions (Bruce et al., 2016). To find strategies for resisting this undesired effect from commercials in food decision-making, we previously tested the feasibility of a food advertising literacy intervention (Ha et al., 2018). This pilot study's results suggested that the food advertising literacy intervention could reduce the relative importance of the taste attribute in food decisions.

Yet, whether the decreased relative importance of taste attributes in food decisions is related to changes in the processing of unhealthy food taste remains unanswered. To validate whether the intervention influences children to process unhealthy foods less tasty, further investigation is necessary. Furthermore, whether an advertising literacy intervention reduces actual snack consumption needs to be examined. In our previous study (Ha et al., 2018), the advertising literacy intervention did not change children's food choices in computerized tasks. Thus, it is important to examine how an advertising literacy intervention would impact actual snack consumption. We primarily focused on unhealthy food taste processing and snack consumption because reducing consumption of tasty but unhealthy foods with high sugar, salt, and fat will have short- and long-term benefits for healthy eating and weight management (Piernas and Popkin, 2010; Ha et al., 2019). In addition, we made a few modifications to test the effectiveness of the intervention with more challenges and control. First, to add the *ad libitum* snack food consumption task, we replaced two commercials that advertised non-fast food restaurants targeting adult consumers (i.e., Chili's^®^ and Applebee's^®^) with new commercials that advertised snack food items targeting children (i.e., Chips Ahoy^®^ and Oreo^®^). This replacement served to test whether the intervention effect would be demonstrated with commercials that specifically target children. Secondly, in our previous work, we randomized group assignments, but the study was not double blind. To ensure the intervention effect was not related to an experimenter bias, further control with a double-blind design was applied. In the present study, to confirm and expand the initial feasibility testing of the food advertising literacy intervention, we tested how the food advertising literacy intervention impacts children's food decision-making focusing on the relative importance of the taste attributes, taste processing of unhealthy and healthy foods, and *ad libitum* snacking in a double-blind intervention procedure. In addition, we speculated children's spontaneous attitudes toward commercials and intervention narratives using the think-aloud method.

We hypothesized that food advertising literacy training would decrease positive attitudes toward commercials in children. We also hypothesized that the food advertising literacy intervention would decrease the susceptibility to unhealthy food decision-making as indicated by (1) the reduced relative decision weights of taste attributes in food decisions, (2) reduced tasty perception and categorization of unhealthy foods, (3) healthier food choices, and (4) decreased amounts of snack food consumption. We expected no such changes among children in the control condition.

## Materials and Methods

### Participants

Thirty-six healthy children (21 girls, 15 boys) aged 8–12 years (*M* = 10.51 years, *SD* = 1.45) with normal or corrected-to-normal vision and hearing participated. Children with a history of neurological conditions, clinically significant psychopathology, or learning disabilities reported by parents were excluded. All participants were recruited from the Kansas City metropolitan and nearby rural areas, and spoke English as their first language. Upon arrival at the laboratory for the first session, a parent gave written informed consent, and a child gave written assent. Then, children's heights and weights were measured in light indoor clothing and stocking feet using a Perspective Enterprises standard stadiometer (PE-WM-60-84; Portage, Michigan) and a Befour scale (PS6600 ST; Saukville, Wisconsin). Body mass index (BMI) scores were converted to age- and sex-specific BMI-for-age percentiles (*M* = 63.82, *SD* = 32.20, range 5.7–99.3). Based on the Centers for Disease Control and Prevention (CDC) guidelines, children's BMI-for-age weight status was categorized as healthy weight (*n* = 23; 64%), overweight (*n* = 4; 8%), or obese (*n* = 10; 28%). Children's pubertal growth was assessed by parent report on the Pubertal Development Scale (Petersen et al., 1988; Carskadon et al., 1993). On average, girls were in mid-pubertal growth (mean PDS score = 2.11, *SD* = 0.73; mean PDS category score = 5.86, *SD* = 2.63), and boys were in early pubertal growth (mean PDS score = 1.52, *SD* = 0.56; mean PDS category score = 4.13, *SD* = 1.36), which reflected a typical pattern that pubertal growth begins earlier for girls than boys (Petersen and Crockett, 1985). There was no significant difference for age, *t*_(34)_ = −0.72, *p* = 0.477, *d* = −0.25, or BMI-for-age percentiles, *t*_(34)_ = −0.60, *p* = 0.550, *d* = −0.21, between girls and boys. Participants consisted of 18 White (50%), 12 Multiracial (33.3%), 4 Black or African American (8.3%), and 3 Hispanic or Latina/o (8.3%).

The sample size was at the expected level (18 ≥ for each group) according to an a *priori* power analysis, based on the effect size (*d* = 0.71, two-tailed) of our previous study tested the feasibility of the advertising literacy intervention in changing children's food decision-making (Ha et al., 2018) with a statistical power of 0.80. Children were randomly assigned to either the intervention condition (*n* = 18; 11 girls, 7 boys; *M* = 10.06 years, *SD* = 1.37; *M* = 57.21th BMI percentile, *SD* = 31.36), or the control condition (*n* = 18; 10 girls, 8 boys; *M* = 12.90 years, *SD* = 1.43; *M* = 70.43th BMI percentile, *SD* = 32.52) after the baseline assessment. The group assignment was double-blinded so that neither participants nor the main experimenter were aware of the group assignment. The age, *t*_(34)_ = 1.91, *p* = 0.065, *d* = 0.66, BMI-for-age percentile, *t*_(34)_ = 1.24, *p* = 0.223, *d* = 0.43, and sex-ratio, $\chi_{\left(1,\text{N}=36\right)}^{2}$ = 0.11, *p* = 0.735, were not significantly different between the two groups. Eleven additional children were recruited but excluded from analysis due to the completion of the first session only (*n* = 5), procedure errors by experimenters (*n* = 3), task non-compliance (a lack of response variety by responding trials with the same response in food ratings and choices; *n* = 2), and not paying attention (whining and crying during the post-intervention session; *n* = 1). This study was approved by the Human Subjects Committee at the University of Kansas Medical Center and approved for a request to rely on the Institutional Review Board of the University of Missouri–Kansas City (FWA00003411). All parents of participants in this study gave written informed consent and child participants gave written assent.

### Procedure

#### Food Advertising Literacy Intervention

##### Pre-intervention

To ensure children's adequate hunger levels for realistic eating choices, children were instructed to fast for 2 h before coming to the laboratory. Upon children's arrivals to the laboratory, the first experimenter measured children's height and weight. To measure the intervention effect, the computerized food rating and choice tasks (Bruce et al., 2016; Ha et al., 2016, 2018, 2019; Lim et al., 2016) were completed at pre-intervention (i.e., before children watching the intervention video at the first session) and post-intervention (i.e., after children watching the intervention video at the last session) in the laboratory. At pre-intervention, the first experimenter asked children to report their hunger levels using an 11-point visual analog scale for hunger (King et al., 1994). Next, the first experimenter instructed children to complete food rating and choice tasks for measuring children's baseline food health and taste ratings and decision weights in food decision-making. To ensure children's motivation for realistic eating choices, children were told that they would receive one of the food items that they selected to eat in the choice task after completing the session, and they received one item from their choices at the end of the session. After completing the baseline measurement, the second experimenter randomly assigned children to one of two groups.

##### Intervention

Research has shown that interventions that utilize narratives for activating advertising literacy successfully increase cognitive skepticism and negative attitudes toward commercials, which reduces susceptibility to the adverse effect of television food advertising in children ages between 8 and 12 years (Buijzen, 2007; Rozendaal et al., 2016; De Jans et al., 2017). Extending our previous work, we administered a food advertising literacy intervention (Ha et al., 2018) to test whether promoting resilience to advertising reduces unhealthy food decision-making.

**Figure 1:**
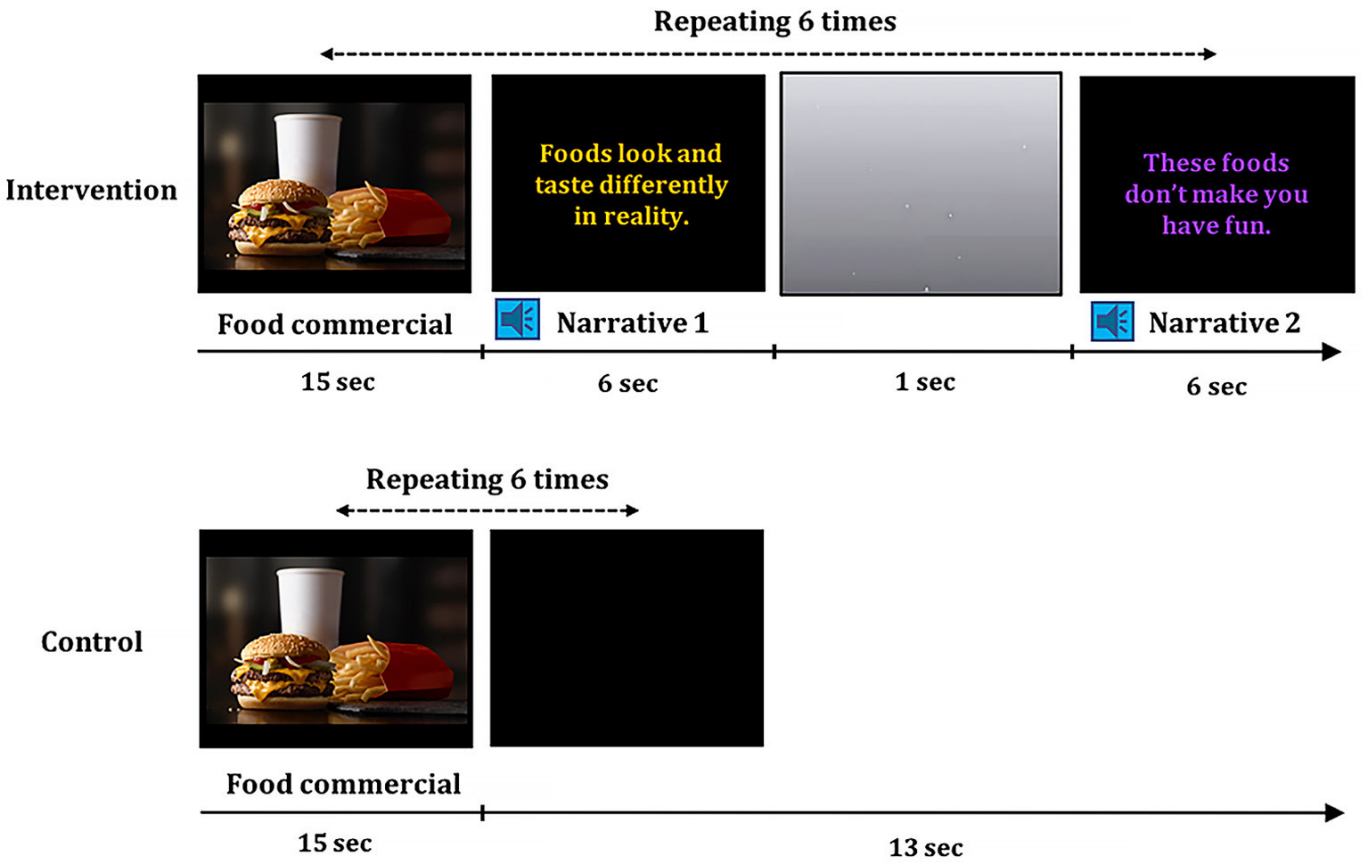
Intervention and control video stimuli. A video for the intervention condition was consisted of six food commercials with embedded 12 factual and evaluative narratives. Each food commercial was followed by two narratives. Narrative statements in colored text moved side-to-side and was accompanied by a female voice. A video for the control session was consisted of the same six food commercials, but no narratives were delivered.

The intervention consisted of a total of twelve factual (cognitive) narratives for enhancing cognitive defenses, i.e., understanding of selling and persuasive intent of advertisers and cognitive skepticism toward television food advertising, and evaluative (affective) narratives for decreasing positive affective attitudes toward television food advertising (Buijzen, 2007; Rozendaal et al., 2012) (see Table 1). The intervention was delivered using a video containing six television food commercials and 12 factual and evaluative narratives (see Figure 1). Each food commercial clip was followed by two narrative statements one-by-one. To help children pay attention to and engage with narratives, a statement in colored text moved side-to-side on the black screen, which was accompanied by an adult female voice reading a statement in child-directed speech. To make each narrative distinctive, an animated video stimulus with small bubbles on a gray screen was presented briefly (1 s) between two narratives. We adopted television commercials for advertising fast food restaurants or unhealthy snack brands (i.e., Chips Ahoy^®^, Denny's^®^, McDonald's^®^, Subway^®^, Oreo^®^, and Wendy's^®^) that were used in a child eating study (Gearhardt et al., 2014), and these commercials were used for our previous work testing children's commercial exposure and food decision-making as well (Bruce et al., 2016; Lim et al., 2016; Ha et al., 2018). Each commercial was 15 s long and the narrative part was presented for ~12 s. In total, two intervention videos were created, and each video used different commercials for the same six brands. The order of food commercials and narratives were pre-randomized for each video. Children watched one of two videos in each session, and the order of videos were counterbalanced across children (e.g., 1212, 2121).

**Table 1:** Narratives for the food advertising literacy intervention.

**Factual (cognitive) narratives**	**Evaluative (affective) narratives**
• Foods look and taste differently in reality.	• These foods don't make you have fun.
• The advertisers want you to go and eat these foods.	• Those foods are disgusting.
• These commercials are intended to sell.	• People in these commercials aren't cool.
• The advertisers are trying to trick you.	• These foods don't make you happy.
• These commercials aren't telling the truth.	• These foods are bad for you.
	• Those foods are not delicious.
	• Those foods are so unhealthy.

Overall, children had four brief intervention sessions over a 1-week period. Because children had to complete the computerized food decision-making tasks that provided the baseline and intervention effect measurements of food decision-making in the laboratory, children visited the laboratory twice during the 1-week period. The first session was done following the baseline measurement at pre-intervention and the fourth session was done before the intervention effect measurement at post-intervention in the laboratory. To ensure children received advertising literacy information frequently, the second and third sessions were done at home with parent assistance. To boost active information processing, children were instructed to speak aloud while watching the intervention video (“think-aloud”). Children completed surveys on advertising knowledge and attitude toward commercials after watching the video in each session. After having the intervention session in the laboratory during the first and fourth sessions, children had an *ad-libitum* snack consumption task.

More specifically, children had the first intervention session in a quiet room in the laboratory. The second experimenter played the video, stepped out of the room, not closing the door all the way and waited in front of the door to encourage children to speak their thoughts out loud while watching a video (“think-aloud”), to prevent the first experimenter across the room from overhearing the audio, and to be able to go back to the room when children needed help. Then children filled out food commercial questionnaires that consisted of multiple short surveys for providing advertising literacy and attitudes toward commercials and the advertised foods (Rozendaal et al., 2012; Gearhardt et al., 2014). While completing the questionnaires, children had an *ad libitum* snack-consumption task (Harris et al., 2009). Lastly, the first experimenter explained instructions for home sessions to a parent. To assist in ensuring the sessions be held without forgetting, the experimenter asked the parent to pick two dates for the home sessions and those dates were written on the instruction document. The parent took the videos saved on a USB flash drive, and food commercial questionnaires for home sessions, which were put in an envelope. Email reminders were sent the day before picked dates.

The second and third sessions were held at home. Children watched one of two videos in each session following pre-counterbalanced order described above. Parents reminded children to think aloud while watching a video, and recorded children's think-aloud vocalizations using apps on smart phones. After watching a video, children completed the food commercial questionnaires in each session.

For the fourth session, children revisited the laboratory at the end of the 1 week period (*M* = 7.21 days, *SD* = 0.51). Children were again instructed to fast for 2 h, and completed the visual analog scale of hunger (*M* = 5.68, *SD* = 2.97). Children watched a video and filled out the food commercial questionnaires.

##### Post-intervention

At post-intervention, right after having the fourth session in the laboratory, children reported their hunger levels using an 11-point visual analog scale for hunger. And then they completed the food rating and choice tasks, which were the same tasks they completed at pre-intervention but in a different, randomized order of trials, to provide food health and taste ratings and decision weights in food decision-making after completing the intervention. Lastly, children had the *ad libitum* snack-consumption task.

#### Control Condition

Similar to the intervention condition, children in the control condition had four control sessions over 1 week (*M* = 7.39 days, *SD* = 0.70). All materials and procedures were identical to the intervention condition except for two control videos that did not include the narratives embedded into commercials, which were replaced with a black screen without any text or sound. Children's hunger levels at the pre-control session in the control group (*M* = 6.07, *SD* = 2.32) were not different from those at pre-intervention in the intervention group (*M* = 5.39, *SD* = 3.10), *t*_(34)_ = 0.74, *p* = 0.466, *d* = 0.25. Children's hunger levels at post-control session in the control group (*M* = 6.45, *SD* = 2.85) were not different from those at post-intervention in the intervention group (*M* = 5.68, *SD* = 2.97), *t*_(33)_ = 0.78, *p* = 0.440, *d* = 0.27.

#### Think-Aloud

Children were instructed that researchers were interested in what they were thinking when they watched the video clip and were encouraged to speak out loud any words that came to mind while watching the video. Spoken responses were recorded. Children's spoken words during each session were coded following the coding scheme (Rozendaal et al., 2012). In particular, spoken words were coded based on relevance of thought (*relevant* to commercials or *irrelevant* to commercials), and origin of thought (*message-originated*, description of commercials or *recipient-generated*, original reactions to commercials). Only relevant, recipient-generated responses were further considered based on (1) nature of thought (*cognitive* beliefs, e.g., “But it's fake” or *affective* responses, e.g., “It's gross!”); (2) polarity of thought (*positive* favorable thoughts, e.g., “That looks so good,” *neutral* thoughts, or *negative* unfavorable thoughts, e.g., “People make bad choices to eat those unhealthy foods”); and (3) advertising understanding (*understanding* or *no understanding* of advertising intentions and tactics) (Rozendaal et al., 2012; Ha et al., 2018). Two research staff members coded children's spoken words independently, and a third research staff member coded disagreed items and finalized coding. We computed Cohen's kappa (*k*) for intercoder reliability for each coding category in each participant. The mean kappa was 0.91 (*SD* = 0.16) for relevance of thought, 0.86 (*SD* = 0.18) for origin of thought, 0.96 (*SD* = 0.06) for nature of thought, 0.96 (*SD* = 0.10) for polarity of thought, and 0.94 (*SD* = 0.04) for advertising understanding. The average interrater agreement was 96.1% (*SD* = 3.5%).

To measure children's cognitive skepticism and critical thinking toward commercials, we computed the ratio of negative cognitive responses, i.e., negative cognitive/(negative cognitive + positive cognitive), and the ratio of negative affective responses, i.e., negative affective/(negative affective + positive affective). A higher negative cognitive response ratio indicated the relatively higher cognitive skepticism and critical thinking toward commercials, and a higher negative affective response ratio indicated the relatively higher negative affective attitudes toward commercials.

#### Questionnaires

##### Food Commercial Questionnaires

We used the modified (1) belief of the commercial scale (2-item; a higher mean score across items indicates higher beliefs for commercials) to measure beliefs toward commercials, (2) liking of the commercial scale (5-item; a higher mean score across items indicates liking of commercials) to measure affective responses toward commercials, and (3) positive attitude toward the brand scale (2-item; a higher mean score across items indicates positive attitudes toward commercials) to measure attitude toward the advertised food (Rozendaal et al., 2012) on 5-point scales (e.g., “not at all” to “very much”). Children provided their responses for each food commercial they watched (a total of six commercials in each session), and the mean value of the six responses represented the score for the specific item. In addition, we measured children's (4) perceived advertising influence on food preferences (1-item; a higher score indicates higher advertising impacts on food preferences), and (5) perceived advertising influence on food choices (1-item; a higher score indicates higher advertising impacts on food choices) on a 5-point scale (“not at all” to “very much”). For the last two scales, responses were not obtained for each food commercial to measure overall perception of advertising impact on their food liking and choices.

#### *Ad libitum* Snack-Consumption Task

While completing questionnaires at the end of the first and last sessions, children were given a total of three plates where each plate had 1.5 servings of Chips Ahoy^®^ cookies (48 grams), Oreo^®^ cookies (51 grams), or Goldfish^®^ crackers (45 grams) based on serving sizes on each snack item's nutrition facts label. Chips Ahoy^®^ and Oreo^®^ cookies were snack items advertised in the videos. Goldfish^®^ crackers was chosen based on the previous study tested children's *ad libitum* snack food consumption, and to make the total amounts of three snack items would be similarly matched to the amount tested in the previous study (Harris et al., 2009). The amounts of food items were measured using an Ozeri Pronto digital multifunction scale. Children were instructed to eat freely at the end of first and fourth sessions, and plates were removed if the child finished all the snacks or after 20 min of eating.

#### Food Rating and Choice Tasks

**Figure 2:**
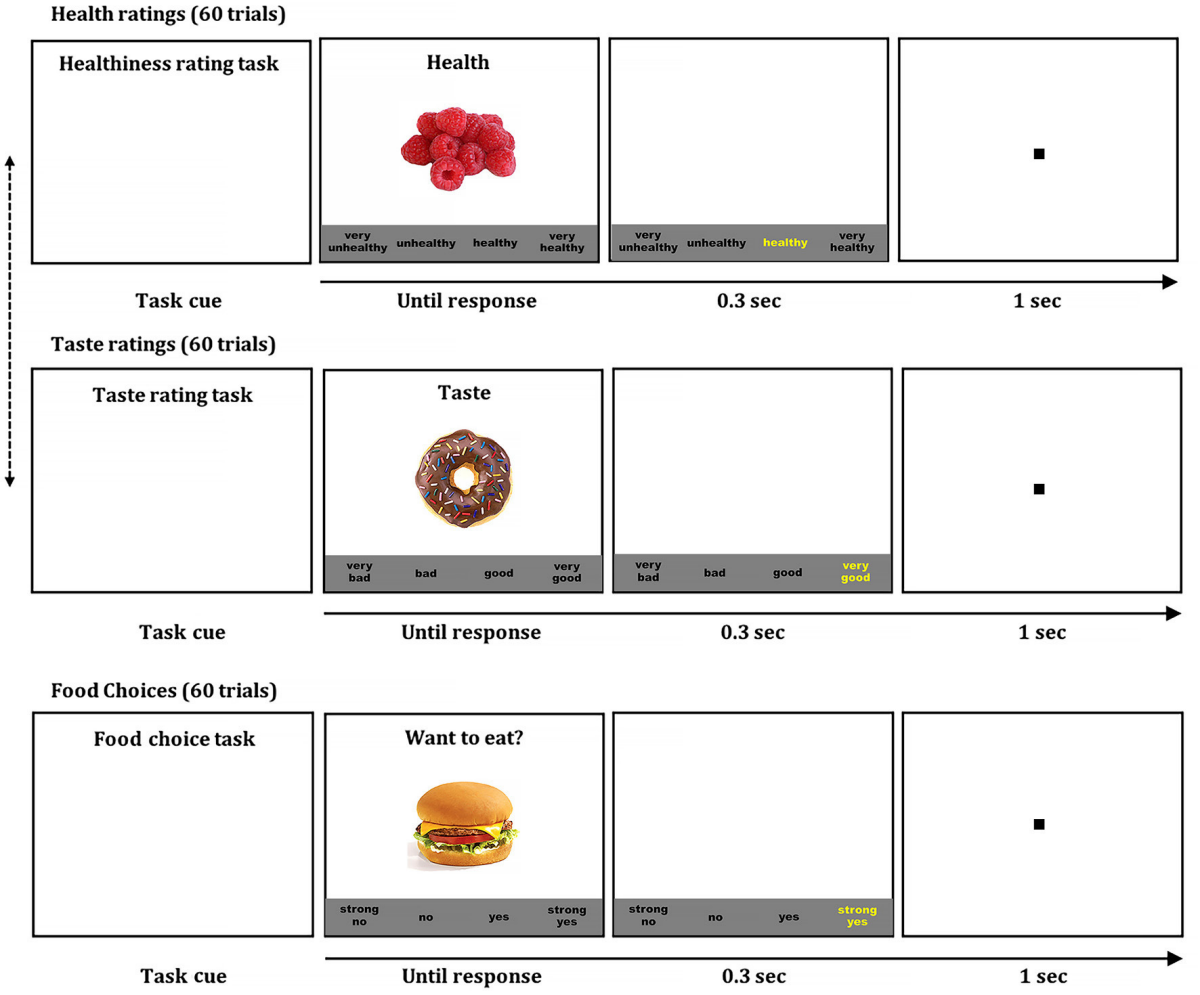
Food ratings and choice tasks. Children rated food healthiness and taste of 60 food items (30 unhealthy and 30 healthy) using four-point scales (health: very unhealthy, unhealthy, healthy, very healthy; taste: very bad, bad, good, very good). Then, children made food decisions on the same 60 food items using a four-point scale (strong no, no, yes, strong yes). Each task began with a task cue. When children pressed a space bar, a colored food image was presented on a white background in the center of the screen that remained on the screen until children made a response, and options of a four-point scale were shown at the bottom. When children chose an option, the selected option was briefly highlighted in yellow to provide visual feedback of their selection. A fixation point was presented for 1 s before the beginning the next trial. The order of food items was randomized in each task, and the order of health and taste ratings were counterbalanced across children.

We measured children's perceived food health and taste attribute ratings, and food choices using computerized tasks (Bruce et al., 2016; Lim et al., 2016; Ha et al., 2018, 2019) (Figure 2). Sixty colored food images with high resolution (72 dpi, 300 X 300 pixels; 30 healthy and 30 unhealthy foods items) were presented one-by-one on a white-background in the center of the screen in a randomized order. Children rated health attributes (“very unhealthy” to “very healthy,” or “very healthy” to “very unhealthy”) and taste attributes (“very bad” to “very good,” or “very good” to “very bad”) for each food item on a 4-point scale by pressing a key on a keyboard. Children were asked to provide health ratings regardless of taste attributes, and taste ratings regardless of health attributes. Health rating and taste rating were measured separately, and the order of two rating tasks was counterbalanced across children. Then children made food choices (“Do you want to eat?”) for each food item on a 4-point scale (“strong no” to “strong yes,” or “strong yes” to “strong no”). Each task began with an initial instruction displaying a specific task under session, and a food image remained on the screen until a response button was pressed. Each trial was separated by a fixation point of 1 s duration. Four-point rating scale options in black text on a gray box were displayed in the bottom center below the food image. When children chose an option, it turned into yellow to provide visual feedback. Presentation^®^ software (version 20; Neurobehavioral Systems, Berkley, California; RRID: SCR_002521) controlled the stimulus presentation and response collection.

### Statistical Analyses

Following our previous statistical analysis model (Bruce et al., 2016; Lim et al., 2016; Ha et al., 2018, 2019) that detected the determinants of children's food decision-making, we computed the decision weights of taste and health attributes in food choices by fitting a linear regression model that taste and health ratings predicted food choices at the individual level. Taste and health ratings were entered in the regression model simultaneously. Each child's estimated regression coefficient of taste attributes indicated the relative decision weights of the taste in food decisions, and an estimated regression coefficient of health attributes indicated the relative decision weights of the healthiness in food decisions.

## Results

### Food Decision-Making

Mean estimated regression coefficients, ratings, and choices are listed in Table 2. To examine the impact of food taste and health attributes on children's food decisions, we conducted *t* tests with the estimated regression coefficients of taste attributes for pre- and post-intervention separately in each group. Taste attributes significantly predicted food decisions for both the intervention group, *t*_(17)_ = 9.38, *p* < 0.001, *d* = 2.21, and the control group, *t*_(17)_ = 13.02, *p* < 0.001, *d* = 3.02, at pre-intervention. Taste attributes significantly predicted food decisions for both the intervention group, *t*_(17)_ = 9.45, *p* < 0.001, *d* = 2.23, and the control group, *t*_(17)_ = 8.77, *p* < 0.001, *d* = 2.07, at post-intervention as well. Similarly, we conducted *t* tests with the estimated regression coefficients of health attributes for pre- and post-intervention separately in each group. Health attributes did not significantly predict food decisions for the intervention group, *t*_(17)_ = 0.68, *p* = 0.508, *d* = 0.16, nor the control group, *t*_(17)_ = 0.06, *p* = 0.955, *d* = 0.07, at pre-intervention. Health attributes did not significantly predict food decisions for the intervention group, *t*_(17)_= 0.28, *p* = 0.78, *d* = 0.07, nor the control group, *t*_(17)_ = −0.34, *p* = 0.737, *d* = −0.08, at post-intervention. These results suggest that children mainly utilize taste information, but not health information, for their food decisions.

**Table 2:** Mean and standard deviations of beta coefficients, ratings, and choices.

**Group**	**Mean estimated regression coefficients** *****β***** **(*****SD*****)**				**Mean taste ratings (*****SD*****)**				**Mean health ratings (*****SD*****)**				**Mean choices (*****SD*****)**			
	**Taste**		**Health**		**Unhealthy foods**		**Healthy foods**		**Unhealthy foods**		**Healthy foods**		**Unhealthy foods**		**Healthy foods**	
	**Pre**	**Post**	**Pre**	**Post**	**Pre**	**Post**	**Pre**	**Post**	**Pre**	**Post**	**Pre**	**Post**	**Pre**	**Post**	**Pre**	**Post**
Intervention	0.75 (0.34)	0.59 (0.27)	0.02 (0.24)	0.01 (0.16)	3.42 (0.35)	3.19 (0.39)	3.01 (0.38)	2.93 (0.46)	1.93 (0.36)	2.01 (0.38)	3.41 (0.29)	3.27 (0.33)	3.19 (0.37)	3.07 (0.48)	2.83 (0.39)	2.86 (0.36)
Control	0.68 (0.22)	0.57 (0.28)	0.003 (0.19)	−0.01 (0.17)	3.33 (0.28)	3.17 (0.33)	2.93 (0.38)	2.87 (0.47)	2.01 (0.53)	2.19 (0.64)	3.22 (0.44)	3.18 (0.52)	3.09 (0.29)	3.09 (0.37)	2.66 (0.37)	2.76 (0.44)

### The Intervention Effect

#### Decision Weights of Taste and Health Attributes

**Figure 3:**
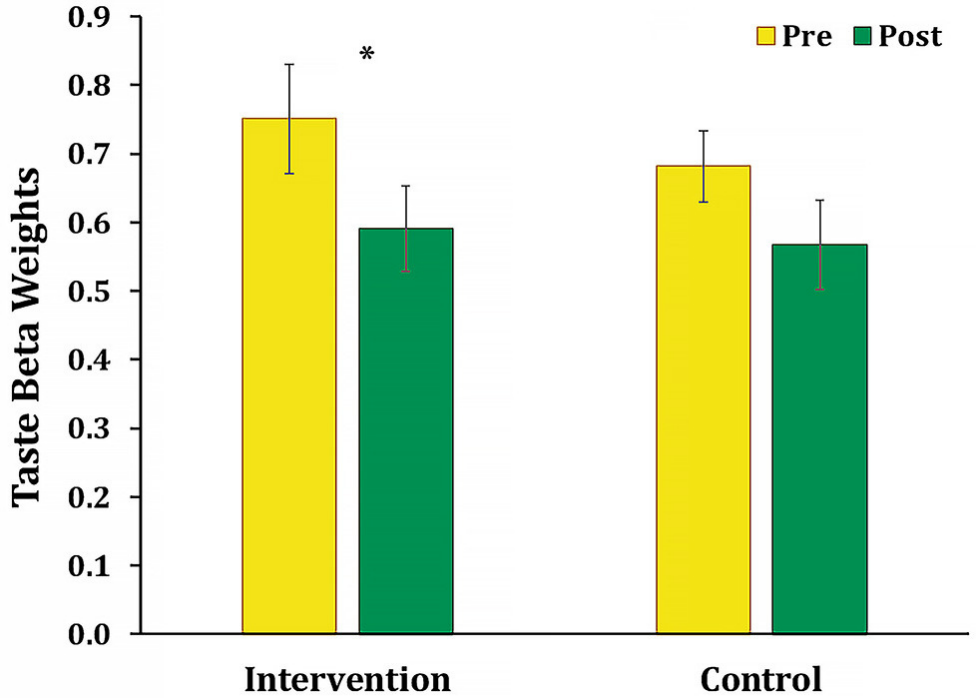
Mean taste beta coefficients in the intervention and control groups. The mean regression beta coefficients of taste attributes was significantly decreased between pre- and post-intervention sessions in the intervention group (^*^p = 0.046). There was no significant change of the mean regression beta coefficients of taste attributes between pre- and post-control sessions in the control group.

To examine the effect of food advertising literacy intervention or control sessions on the relative decision weights of taste attributes, we compared the mean estimated regression coefficient of taste attributes between pre- and post-session within each group. Planned comparisons revealed that the estimated regression coefficient of taste attributes was significantly decreased in the intervention group after completing the intervention, *t*_(17)_ = 2.15, *p* = 0.046, *d* = 0.51, which confirmed the hypothesis 1 (see Figure 3). In contrast, the estimated regression coefficient of taste attributes was not significantly changed after completing control sessions in the control group, *t*_(17)_ = 1.65, *p* = 0.118, *d* = 0.39. We also examined the effect of food advertising literacy intervention or control condition on the relative decision weights of the healthiness within each group. The estimated regression coefficient of health attributes was not significantly after completing the intervention in the intervention group, *t*_(17)_ = 0.45, *p* = 0.661, *d* = 0.11, nor after completing the control sessions in the control group, *t*_(17)_ = 0.48, *p* = 0.639, *d* = 0.11. These results suggest that the advertising literacy intervention effectively reduces the relative importance of the taste in children's food decisions, which was not observed in the control condition. It is noteworthy to mention that these results replicated our previous work testing the feasibility of the food advertising literacy intervention (Ha et al., 2018).

#### Taste and Healthiness Perceptions

To explore the effect of intervention or control sessions on food taste and healthiness perceptions, we compared the mean taste and health ratings of unhealthy (30 food items) and healthy foods (30 food items) separately between pre- and post-session within each group. For the intervention group, comparisons demonstrated that unhealthy foods taste ratings significantly decreased after completing the intervention, *t*_(17)_ = 2.55, *p* = 0.021, *d* = 0.60, whereas unhealthy foods health ratings were not changed, *t*_(17)_ = −1.43, *p* = 0.171, *d* = −0.34. Healthy foods taste ratings, *t*_(17)_ = 0.91, *p* = 0.376, *d* = 0.21, and healthy foods health ratings, *t*_(17)_ = 1.73, *p* = 0.102, *d* = 0.41, were not significantly changed. These results suggest that children perceive unhealthy foods as less tasty after receiving the intervention. For the control group, unhealthy foods taste ratings significantly decreased, *t*_(17)_ = 2.47, *p* = 0.024, *d* = 0.58, meanwhile unhealthy foods health ratings significantly increased, *t*_(17)_ = −2.79, *p* = 0.013, *d* = −0.66, after completing the control sessions. Healthy foods taste ratings, *t*_(17)_ = 0.85, *p* = 0.408, *d* = 0.20, and healthy foods health ratings, *t*_(17)_ = 0.79, *p* = 0.439, *d* = 0.19, were not significantly changed. These results suggest that children in the control condition, who were exposed to food commercials without intervention, show an adverse effect of evaluating unhealthy foods healthier. Considering the association between unhealthiness and tastiness (Raghunathan et al., 2006; Ha et al., 2019), perceiving unhealthy foods less tasty could be linked to adverted food healthiness evaluations.

#### Tasty Categorization

To confirm the intervention effect of perceiving unhealthy foods less tasty, we compared tasty categorizations of unhealthy (30 food items) and healthy foods (30 food items) separately between pre- and post-session within each group. Based on children's taste ratings, food items were categorized as *tasty* (i.e., “good” or “very good” ratings), or *not-tasty* (i.e., “bad” or “very bad” ratings), for unhealthy and healthy foods separately. Then, we examined the percentages of *tasty* and *not-tasty* food items for unhealthy foods. In the intervention group, the percentages of *unhealthy/tasty* food items significantly decreased from pre-intervention (*M* = 90.9%, *SD* = 9.1) to post-intervention (*M* = 84.6%, *SD* = 12.7), *t*_(17)_ = 2.52, *p* = 0.022, *d* = 0.59, which also reflected the significant increase of the percentages of *unhealthy/non-tasty* foods items from pre-intervention (*M* = 9.1%, *SD* = 9.1) to post-intervention (*M* = 15.4%, *SD* = 12.7), *t*_(17)_ = −2.52, *p* = 0.022, *d* = −0.59, which confirmed the hypothesis 2. In the control group, there was no significant changes of the percentages of *unhealthy/tasty* food items between pre-control session (*M* = 90.2%, *SD* = 10.5) and post-control session (*M* = 87.0%, *SD* = 12.7), *t*_(17)_ = 1.40, *p* = 0.179, *d* = 0.33, which reflected no significant percentage changes of *unhealthy/non-tasty* foods items between pre-control session (*M* = 9.8%, *SD* = 10.5%) and post-control session (*M* = 13.0%, *SD* = 12.7%), *t*_(17)_ = −1.40, *p* = 0.179, *d* = −0.33. These results suggest that the intervention influences children to perceive unhealthy foods less tasty.

Additionally, we compared the percentages of *tasty* and *non-tasty* food items for healthy foods between pre- and post-session within each group. In the intervention group, there was no significant percentage changes of *healthy/tasty* foods items between pre-intervention (*M* = 75.4%, *SD* = 17.2) and post-intervention (*M* = 73.1%, *SD* = 19.8), *t*_(17)_ = 0.74, *p* = 0.473, *d* = 0.17, which reflected no significant percentage changes of *healthy/not-tasty* foods items between pre-intervention (*M* = 24.6%, *SD* = 17.2) and post-intervention (*M* = 26.9%, *SD* = 19.8), *t*_(17)_ = −0.74, *p* = 0.473, *d* = −0.17. In the control group, there was no significant percentage changes of *healthy/tasty* foods between pre-control session (*M* = 74.8%, *SD* = 16.7%) and post-control session (*M* = 70.9%, *SD* = 19.2), *t*_(17)_ = 1.51, *p* = 0.149, *d* = 0.36, which reflected no significant percentage changes of *healthy/not-tasty* foods items between pre-control session (*M* = 25.2%, *SD* = 16.7%) and post-control session (*M* = 29.1%, *SD* = 19.2), *t*_(17)_ = −1.51, *p* = 0.149, *d* = −0.36.

#### Attitudes Toward Commercials

We examined children's beliefs, liking, positive attitudes toward commercials as well as advertising impact on food preferences and food choices using children's self-report on food commercial questionnaires for each group (see Supplementary Table 1 for descriptive statistics). For the intervention group, children's perceived advertising influence on food preferences significantly decreased between the first session and the last session, *t*_(17)_ = 2.32, *p* = 0.033, *d* = 0.55, suggesting that children perceived food commercials as having less impact on their food preferences after completing the intervention. For the control group, the liking of the commercial significantly decreased between the first session and the last session, *t*_(17)_ = 2.69, *p* = 0.016, *d* = 0.63, suggesting that children perceived food commercials they watched as less likable after completing the control sessions. Results of other attitudes measured using food commercial questionnaires were not significant.

**Figure 4:**
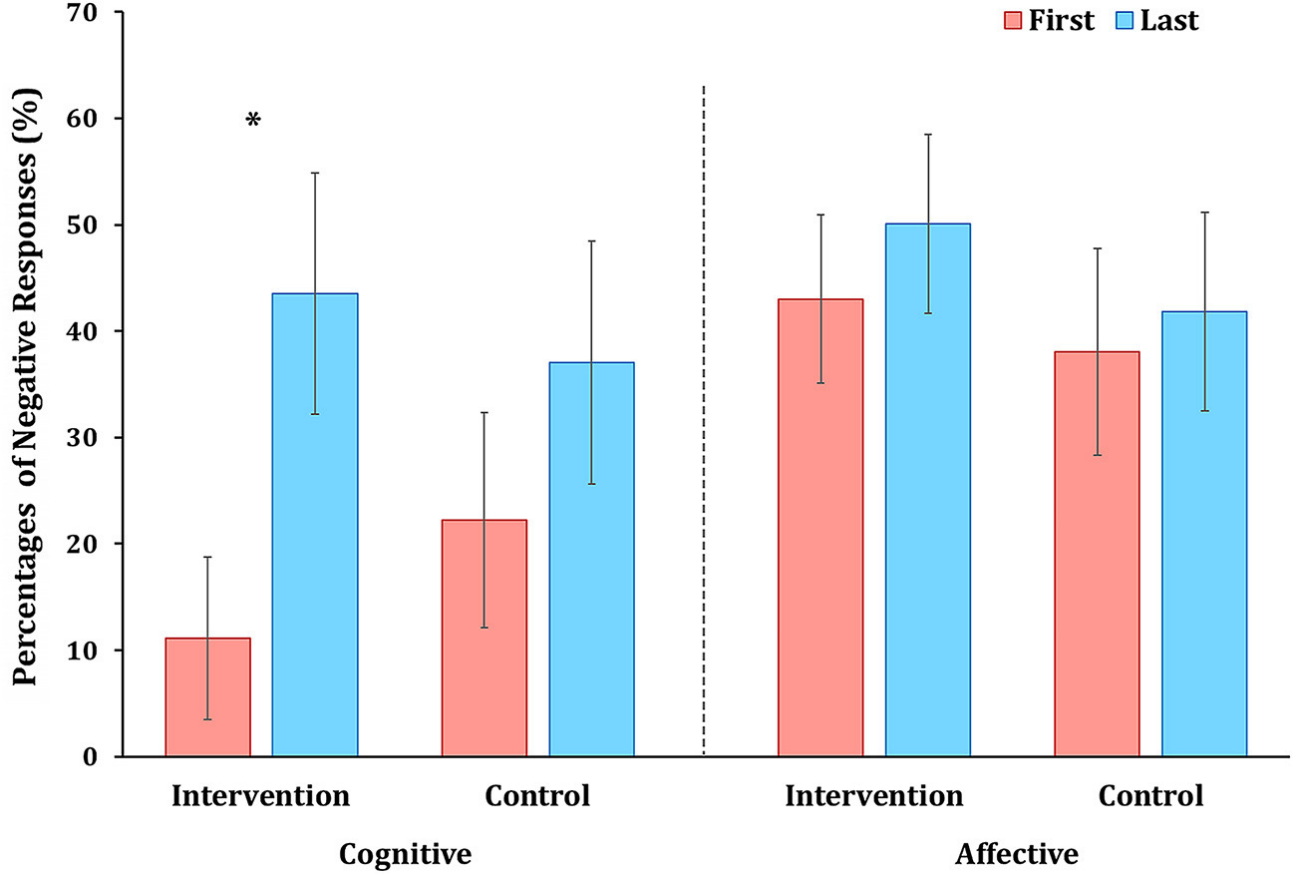
Mean percentages of negative cognitive and affective responses toward commercials. The mean percentage of relative negative cognitive toward commercials was significantly increased between the first and last sessions in the intervention group (^*^p = 0.016), while no significant change was found in the control group. There were no significant changes of relative negative affective responses in both groups.

To further investigate how the intervention influenced children's cognitive skepticism and critical thinking and affective responses toward commercials at the time of exposure, we examined children's spoken thoughts while watching food commercials obtained using the think-aloud method (see Supplementary Table 2 for descriptive statistics). During four sessions, children showed more affective responses than cognitive responses relevant to food commercials while watching commercials in the intervention group, *t*_(17)_ = 3.86, *p* < 0.001, *d* = 0.91, as well as in the control group, *t*_(17)_ = 4.10, *p* < 0.001, *d* = 0.99. The percentages of negative cognitive responses estimated the relative cognitive skepticism and critical thinking toward commercials in cognitive responses, and the percentages of negative affective responses estimated the relative negative affective attitudes toward commercials in affective responses. For the intervention group, the percentages of negative cognitive responses toward commercials were significantly increased from the first session to the last session, *t*_(17)_ = −2.68, *p* = 0.016, *d* = −0.63, but the percentages of negative affective responses toward commercials were not significantly different between the first session and the last session, *t*_(17)_ = −0.89, *p* = 0.388, *d* = −0.21 (see Figure 4). For the control group, the percentages of negative cognitive responses toward commercials were not significantly different between the first session and the last session, *t*_(17)_ = −1.19, *p* = 0.0249, *d* = −0.28, nor the percentages of negative affective responses toward commercials were not significantly different between the first session and the last session, *t*_(17)_ = −0.38, *p* = 0.710, *d* = −0.21. These results suggest that the intervention effectively enhances children's cognitive skepticism and critical thinking toward commercials.

#### Opinions on Advertising Literacy Narratives

To explore children's cognitive and affective attitudes toward intervention narratives, we examined children's spoken words while listening to narratives between commercials using the think-aloud method in the intervention group. The percentages of negative cognitive responses toward narratives were not significantly different between the first session and the last session, *t*_(17)_ = −1.89, *p* = 0.077, *d* = −0.45. The percentages of negative affective responses toward narratives were not significantly different between the first session and the last session, *t*_(17)_ = −1.14, *p* = 0.270, *d* = −0.27.

We observed that children often expressed opinions about the narratives. We coded children's spoken words relevant to narratives as disagreement (e.g., “It's your opinion.”; “Don't lie.”), neutral (e.g., “confused”; “I didn't know that.”), and agreement (e.g., “That's true.”; “exactly”) opinions about narratives. We computed the percentages of disagreement (*M* = 44.3%, *SD* = 32.8), neutral (*M* = 8.6%, *SD* = 15.9), and agreement (*M* = 36.0%, *SD* = 33.7) across sessions within each individual. Then, we explored how children's overall opinions to narratives were related to changes in cognitive and affective attitudes toward commercials between the first and the last sessions (i.e., the percentages of cognitive or affective responses at the last session—the percentages of cognitive or affective responses at the first session). The percentages of disagreement to narratives significantly predicted the change in the percentages of negative cognitive responses toward commercials, *b* = −0.758, *SD* = 0.34, *t*_(16)_ = −2.22, *p* = 0.041, *R*^2^ = 0.235. As the relative disagreement to narratives decreased, the relative negative cognitive responses toward commercials increased. This finding suggests that as children accept advertising literacy knowledge over the intervention period, their cognitive skepticism and critical thinking toward commercials were enhanced.

#### Food Choices and *ad libitum* Snacking

To explore the intervention effect on food choices, we compared the mean food choices of unhealthy and healthy foods separately between pre- and post-session within each group. For the intervention group, there was no significant changes in unhealthy food choices between pre-intervention (*M* = 3.19, *SD* = 0.37) and post-intervention (*M* = 3.07, *SD* = 0.48), *t*_(17)_ = 1.33, *p* = 0.200, *d* = 0.31, nor in healthy food choices between pre-intervention (*M* = 2.83, *SD* = 0.39) and post-intervention (*M* = 2.86, *SD* = 0.36), *t*_(17)_ = −0.49, *p* = 0.630, *d* = −0.12, which did not confirm the hypothesis 3. Similarly, for the control group, there was no significant changes in unhealthy food choices between pre-control session (*M* = 3.09, *SD* = 0.29) and post-control session (*M* = 3.09, *SD* = 0.37), *t*_(17)_ = 0.11, *p* = 0.912, *d* = 0.03, nor in healthy food choices between pre-control session (*M* = 2.66, *SD* = 0.37) and post-control session (*M* = 2.76, *SD* = 0.44), *t*_(17)_ = −1.80, *p* = 0.090, *d* = −0.42.

Then, we compared the percentages of children's self-regulated decisions between pre- and post-session. Self-regulated decisions were made when children successfully resisted eating tasty but unhealthy food items (i.e., “no” or “strong no” decisions for *unhealthy/tasty* food items) and chose to eat not-tasty but healthy food items (i.e., “yes” or “strong yes” decisions for *healthy/not-tasty* food items) (Ha et al., 2016; Lim et al., 2016). For the intervention group, there were no significant changes in the percentages of self-regulated decisions between pre- (*M* = 13.5%, *SD* = 12.4%) and post-intervention (*M* = 18.0%, *SD* = 19.5%), *t*_(17)_ = −1.49, *p* = 0.156, *d* = −0.36. Similarly, for the control group, there were no significant changes in the percentages of self-regulated decisions between pre- (*M* = 9.71%, *SD* = 11.0%) and post-intervention (*M* = 12.5%, *SD* = 9.80%), *t*_(17)_ = −1.41, *p* = 0.177, *d* = −0.34.

Further, we examined the relations between the attitude toward commercials and food choices. The increase in negative cognitive responses toward commercials observed during think-aloud between first- and last-sessions predicted a concomitant increase in the percentage of self-regulated decisions between pre- and post-intervention in the intervention group, *b* = 0.120, *SD* = 0.05, *t*_(16)_ = 2.21, *p* = 0.042, *R*^2^ = 0.234. In contrast, in the control group, the increase of relative negative cognitive responses toward commercials did not significantly predict the increase of the percentages of self-regulated decisions, *b* = −0.002, *SD* = 0.04, *t*_(16)_ = −0.06, *p* = 0.956, *R*^2^ = 0.0002. These findings suggest that as children's cognitive skepticism and critical thinking increased, self-regulated decisions increased.

Lastly but importantly, we examined food consumption behaviors for each group to test ecological validity of the intervention effect. The amounts of snack consumption were not significantly different between the first session (*M* = 93.29g, *SD* = 28.63) and the last session in the intervention group (*M* = 101.88g, *SD* = 57.48), *t*_(16)_ = −0.72, *p* = 0.484, *d* = −0.17, which did not confirm the hypothesis 4. Also, the amounts of snack consumption were not significantly different between the first session (*M* = 68.56g, *SD* = 36.64) and the last session in the control group (*M* = 62.22g, *SD* = 39.53), *t*_(17)_ = 1.10, *p* = 0.287, *d* = 0.26. These findings suggest that the advertising literacy training did not change the amount of snack food consumption.

## Discussion

In this study, we examined how enhanced resilience to the adverse effect of food commercials influenced susceptibility to unhealthy food decision-making in children ages 8–12 years. For promoting resilience to food commercials, we utilized the food advertising literacy intervention—four-session, 1-week intervention held in both the laboratory and home environment (Ha et al., 2018). This intervention was intended to improve cognitive and affective defenses against food advertising by delivering factual and evaluative narratives. Indeed, children demonstrated higher cognitive skepticism and critical thinking toward the advertising tactics and the advertised foods after completing the intervention, and perceived advertising influence less on their food liking. As hypothesized, the food advertising literacy intervention reduced susceptibility to unhealthy food decision-making. For children who received the intervention, the relative decision weights of taste attributes were significantly decreased in their food decisions, which replicated results of our pilot study (Ha et al., 2018). In addition, children categorized lower number of unhealthy food items as tasty (i.e., *unhealthy/tasty*) after completing the intervention, which suggests reduced tasty processing of unhealthy foods. The results based on changes between the baseline and the completion of intervention suggest that the rate of food choices or the amounts of snack consumption were not changed. But, the speculation on the relations between children's attitudes toward commercials and food decision-making demonstrates that as children's cognitive defenses toward commercials enhanced, their self-regulated decisions are increased. These findings may suggest the advertising literacy intervention is effective in enhancing self-regulated eating decisions as children's cognitive defenses improves.

In contrast, for children in the control condition, children did not show changes in cognitive skepticism and critical thinking toward commercials. Regarding the susceptibility to unhealthy food decision-making, the relative decision weights of taste attributes were not significantly changed. Children perceived unhealthy foods as less tasty after completing the control sessions similar to children in the intervention condition. However, considering that they evaluated unhealthy foods healthier than before, decreased taste perception of unhealthy foods could be related to the adversely evaluated food healthiness. Children in the control condition reported a decreased liking of commercials they watched. Repetitive exposure to the same commercials might decrease liking for those commercials, yet, children's decision weights of taste attributes nor cognitive skepticism and critical thinking toward food commercials were not changed. Actual food choices or amounts of snack food consumptions were not changed neither.

Taken together, findings provide evidence that promoting resilience to food commercials by increasing cognitive skepticism and critical thinking toward food commercials reduce children's susceptibility to unhealthy food decision-making. Given the pervasive advertising effect on heightened attentional vigilance to food brand logos (Masterson et al., 2019b), biased taste preference to branded foods (Robinson et al., 2007), and increased liking of fast food even with the exposure to commercials featuring “healthier” fast food meal options (Boyland et al., 2015), the results of this study provide a valuable understanding of strategies for building children's resilience to adverse effects of food advertising. When considering that exposure to food commercials increases the importance of the taste attribute in food decisions (Bruce et al., 2016), the reversed, intervention effect that decreased the importance of the taste attribute in food decisions in this study emphasizes the benefits of cognitive defenses in combating undesired effects of food commercials and taste-oriented, unhealthy eating decisions.

The brief, 1-week intervention was not sufficient to change the amount of actual snack food consumption. Since snack items used in the *ad libitum* task were familiar branded snack food items (Keller et al., 2012), and television food advertising has a strong priming effect in increasing snack consumption in children (Harris et al., 2009; Russell et al., 2019), more pervasive intervention tactics may be required to change actual amounts of food consumption. One neuroimaging study that tested the influence of television food commercials on food consumption reported that watching food commercials did not significantly change the amounts of meal consumption in the laboratory, however, exposure to high-energy food items reduced brain activations in the prefrontal cortex that is involved in cognitive control in children who watched food commercials (Masterson et al., 2019a). Thus, future studies should examine how the advertising literacy intervention would influence children's brain responses to food commercials and unhealthy and healthy food decision-making and implications for actual food consumption when both healthy and unhealthy options are provided.

Additionally, there is a possibility that when and who delivers the narratives, and what specific contents are targeted matter in a food advertising literacy intervention. When Rozendaal and her colleagues (2016) created an animation character to deliver the factual narratives prior to commercials only in one session, children who had a narrative targeting manipulative intent of the advertising as a forewarning showed more negative affective attitudes toward commercials that led to lower desire to the advertised product compared to comparison groups (Rozendaal et al., 2016). Still, it is unknown whether the forewarning method may reduce the relative importance of taste attribute in food decisions.

Another important aspect to address is the advantage of a “think-aloud” method for speculating children's information processing online. The think-aloud method allowed us to explore children's spontaneous responses at the moments of food commercial exposure (Rozendaal et al., 2012). In this study, the intervention effect was related to the changes in cognitive defenses toward commercials. Unexpectedly, we observed that children often expressed opinions about the intervention narratives, and those thoughts reflected children's own perspectives and attitudes toward narratives and advertised foods. As assimilation of advertising literacy occurred, children's cognitive skepticism and critical thinking toward commercials increased. These findings suggest that especially for those children with opponent thoughts to narratives, interactive learning providing more explanations for advertising tactics and healthier food options could be helpful to enhance defenses against food advertising.

The think-aloud process itself could be effective in activating advertising literacy. From the perspective of the development of information processing that children retrieve and apply knowledge when cues are provided, the think-aloud process could act as a cue for activating cognitive defenses that decrease susceptibility to advertising (Rozendaal et al., 2012). In the present study, children in the control condition who used think-aloud with no intervention, showed a decreased liking of commercials. This decrease could be an effect of the think-aloud method. However, our findings suggest that the think-aloud method was not sufficient to decrease the susceptibility to advertising in that no changes were observed in cognitive skepticism and critical thinking. Moreover, think-aloud itself was not effective in reducing susceptibility to unhealthy food decision-making in that these children did not demonstrate changes in the importance of taste attributes in food decision-making, tasty categorization of *unhealthy/tasty* food items, and food choices. Future studies should examine how the think-aloud method alone and the combined narratives and think-aloud would differently impact susceptibility to commercials and unhealthy food decision-making when they are compared to a passive viewing control group.

Together, the findings of the present study suggest that cognitive defenses to advertising are activated more effectively when children are cued with narratives, and actively utilize and exercise advertising literacy information internally to deflate the undesired influence of advertising while watching food commercials. Successful defenses could be extended to reduce susceptibility to unhealthy food decision-making. In a normal viewing situation, children are less likely to exercise advertising literacy defenses on their own without external narrative cues and encouragement for active application of advertising literacy information (Brucks et al., 1988). Thus, the role of parents is highly important to teach advertising literacy and encourage children to utilize cognitive defenses actively, instead of passively receiving the advertising information, until they develop the internalized and autonomous strategies to overcome the adverse effects of food commercials (Buijzen and Valkenburg, 2005; Buijzen, 2009). A similar environment that the food advertising literacy intervention provides to promote resilience to advertising and reduce susceptibility to unhealthy eating could be built at home or weight-management clinics by having educational conversations or intervention sessions. Education would be especially helpful when parents and children are watching food commercials together. Parental roles are also critical in the development of healthy eating. Our recent work shows that children can make more self-regulated eating decisions while thinking “what my parents would want me to eat,” compared to when they make eating decisions while thinking “what I like to eat” (Lim et al., 2016). These results suggest that parents' guidance on healthy eating could cue and activate self-regulated eating decisions until children's dietary self-control is internalized. Parents should set good examples for their children by exercising controls on their consumer and eating behaviors (Pettigrew et al., 2013).

School and media also need to engage in teaching advertising literacy and healthy food choices more actively so that children practice and build strategies to combat the advertising effect and reduce susceptibility to unhealthy food choices, given that food advertising and unhealthy eating habits are among the major contributing factors of childhood obesity (Kelly et al., 2010). Regulations that intended to limit unhealthy food marketing on television have not been successful in controlling exposure to unhealthy and fast food advertisements in many countries (Campos et al., 2016; Vandevijvere et al., 2017; Whalen et al., 2019). Regulating food commercials is not limited to television advertising anymore since children are exposed to food commercials through various online platforms and smartphone applications that use both direct and indirect methods (Nelson, 2018). Despite growing challenges, advertising literacy interventions have shown promise in promoting resilience to advertising in various formats (Hudders et al., 2016; De Jans et al., 2017). Thus, our society, as a whole, including advertisers and legislators, needs to focus on developing more critical strategic regulations and solutions utilizing advertising literacy, such as an embedded warning in text, especially for high-caloric, low-nutrient unhealthy foods, to regulate children's exposure to unhealthy food commercials. Efforts could include limiting advertising contents and tactics targeting children that could easily sway their attention and trigger affective responses, which could overwhelm children's developing cognitive defenses and provoke overconsumption of unhealthy foods that increase risks of developing obesity (De Jans et al., 2019).

Future studies should investigate an expanded intervention, such as a more active learning opportunity for a prolonged period, could effectively promote both negative affective and negative cognitive attitudes toward commercials that lead to changes of the decision weights of taste attribute, food choices, and food consumption with a larger scale (Hudders et al., 2016; De Jans et al., 2017). To examine whether factual and evaluative narratives influence susceptibility to unhealthy food decision-making differently, future studies should test the intervention effect of factual narratives and evaluative narratives separately. Although the sessions were held frequent, our 1-week brief intervention sessions with narratives did not have enough impact to change the actual rates of food choices and the amounts of snack consumption, similar to our previous study (Ha et al., 2018). Futures studies should further investigate how well the food advertising literacy intervention could be applied for enhancing resilience to food commercials featured in various formats including YouTube videos, social media, and mobile games, targeting children and adolescents. Additionally, considering the relations between unhealthy food decision-making and children's self-control development (Ha et al., 2016, 2019), and the improved self-regulated decisions along with the enhanced cognitive defenses, future studies should address how children's dietary self-control influences the relation between resilience to food commercials and susceptibility to unhealthy food decision-making. Lastly, future studies could utilize a think-aloud method for food choices (Ogden and Roy-Stanley, 2020), which could reveal children's thinking process underneath food decision-making.

This study has several limitations. The sample size was relatively modest. However, in the present study, we replicated the main finding of Ha et al. (2018) that children's relative decision weights of taste attributes decrease after intervention. Across our two studies that report the intervention effect, the probability of committing two consecutive type I error is reduced to 0.0025 or 0.25% (0.05 × 0.05). The effect size of the main intervention effect on the reduced taste importance is *d* = 0.51, which indicates a medium effect size. We believe that replication of previous findings with a medium effect size in the present study could reduce the possibility of false positive and negative observations. The brief 1-week intervention we used did not change the amount of snack food consumption. The findings of this study expand our understanding of the efficacious strategies of promoting resilience to undesired effects of food commercials, which could establish healthy eating habits and less taste-oriented food decisions in children. Furthermore, the findings of this study imply that helping children to build developmentally-suited, defense mechanisms for various external food cues could be effective for prevention and intervention for childhood obesity.

## Data Availability Statement

The raw data supporting the conclusions of this article will be made available by the authors, without undue reservation.

## Ethics Statement

The studies involving human participants were reviewed and approved by the Human Subjects Committee at the University of Kansas Medical Center and the Institutional Review Board at the University of Missouri-Kansas City. Written informed consent to participate in this study was provided by the participants' legal guardian/next of kin.

## Author Contributions

O-RH and AB contributed to the conception and design of the study. O-RH and HK organized the database. HK, JS, and SN collected the data. HK, SN, and O-RH performed the coding. O-RH performed the statistical analysis and wrote the first draft of the manuscript. AB, AD, and S-LL contributed to the discussion of the results and implications. All authors contributed to manuscript revision, read, and approved the submitted version.

## Conflict of Interest

The authors declare that the research was conducted in the absence of any commercial or financial relationships that could be construed as a potential conflict of interest.

## References

[B1] Adise S. Geier C. F. Roberts N. J. White C. N. Keller K. L. (2018). Is brain response to food rewards related to overeating? A test of the reward surfeit model of overeating in children. Appetite 128, 167–179. 10.1016/j.appet.2018.06.014 29890186 PMC7482544

[B2] Beauchamp G. K. Mennella J. A. (2009). Early flavor learning and its impact on later feeding behavior. J. Pediatr. Gastroenterol. Nutr. 48, S25–S30. 10.1097/MPG.0b013e31819774a5 19214055

[B3] Birch L. L. Fisher J. O. (1998). Development of eating behaviors among children and adolescents. Pediatrics 101, 539–549. 12224660

[B4] Blosser B. J. Roberts D. F. (1985). Age differences in children's perceptions of message intent: responses to TV news, commercials, educational spots, and public service announcements. Commun. Res. 12, 455–484. 10.1177/009365085012004002

[B5] Boyland E. J. Halford J. C. (2013). Television advertising and branding. Effects on eating behaviour and food preferences in children. Appetite 62, 236–241. 10.1016/j.appet.2012.01.032 22421053

[B6] Boyland E. J. Harrold J. A. Kirkham T. C. Corker C. Cuddy J. Evans D.. (2011). Food commercials increase preference for energy-dense foods, particularly in children who watch more television. Pediatrics 128, e93–e100. 10.1542/peds.2010-1859 21708808

[B7] Boyland E. J. Kavanagh-Safran M. Halford J. C. (2015). Exposure to ‘healthy’fast food meal bundles in television advertisements promotes liking for fast food but not healthier choices in children. Br. J. Nutr. 113, 1012–1018. 10.1017/S0007114515000082 25716646

[B8] Boyland E. J. Nolan S. Kelly B. Tudur-Smith C. Jones A. Halford J. C.. (2016). Advertising as a cue to consume: a systematic review and meta-analysis of the effects of acute exposure to unhealthy food and nonalcoholic beverage advertising on intake in children and adults, 2. Am. J. Clin. Nutr. 103, 519–533. 10.3945/ajcn.115.120022 26791177

[B9] Bruce A. S. Bruce J. M. Black W. R. Lepping R. J. Henry J. M. Cherry J. B. C.. (2014). Branding and a child's brain: an fMRI study of neural responses to logos. Soc. Cogn. Affect. Neurosci. 9, 118–122. 10.1093/scan/nss109 22997054 PMC3871732

[B10] Bruce A. S. Pruitt S. W. Ha O. R. Cherry J. B. Smith T. R. Bruce J. M.. (2016). The influence of televised food commercials on children's food choices: evidence from ventromedial prefrontal cortex activations. J. Pediatr. 177, 27–32 e21. 10.1016/j.jpeds.2016.06.067 27526621 PMC5242233

[B11] Brucks M. Armstrong G. M. Goldberg M. E. (1988). Children's use of cognitive defenses against television advertising: a cognitive response approach. J. Consum. Res. 14, 471–482. 10.1086/209129

[B12] Buijzen M. (2007). Reducing children's susceptibility to commercials: mechanisms of factual and evaluative advertising interventions. Media Psychol. 9, 411–430. 10.1080/15213260701291361

[B13] Buijzen M. (2009). The effectiveness of parental communication in modifying the relation between food advertising and children's consumption behaviour. Br. J. Dev. Psychol. 27, 105–121. 10.1348/026151008X334719 19972665

[B14] Buijzen M. Valkenburg P. M. (2005). Parental mediation of undesired advertising effects. J. Broadcast. Electron. Media 49, 153–165. 10.1207/s15506878jobem4902_1

[B15] Burton S. Lichtenstein D. R. (1988). The effect of ad claims and ad context on attitude toward the advertisement. J. Advert. 17, 3–11. 10.1080/00913367.1988.10673098

[B16] Campos D. Hernández-Torres J. J. Agil A. Comino M. López J. C. Macías V.. (2016). Analysis of food advertising to children on Spanish television: probing exposure to television marketing. Archiv. Med. Sci. 12:799. 10.5114/aoms.2016.60969 27478462 PMC4947627

[B17] Carskadon M. A. Vieira C. Acebo C. (1993). Association between puberty and delayed phase preference. Sleep 16, 258–262. 10.1093/sleep/16.3.258 8506460

[B18] De Cosmi V. Scaglioni S. Agostoni C. (2017). Early taste experiences and later food choices. Nutrients 9:107. 10.3390/nu9020107 28165384 PMC5331538

[B19] De Jans S. Hudders L. Cauberghe V. (2017). Advertising literacy training: the immediate versus delayed effects on children's responses to product placement. Eur. J. Market. 51, 2156–2174. 10.1108/EJM-08-2016-0472

[B20] De Jans S. Van de Sompel D. Hudders L. Cauberghe V. (2019). Advertising targeting young children: an overview of 10 years of research (2006–2016). Int. J. Advert. 38, 173–206. 10.1080/02650487.2017.1411056

[B21] Enax L. Weber B. Ahlers M. Kaiser U. Diethelm K. Holtkamp D.. (2015). Food packaging cues influence taste perception and increase effort provision for a recommended snack product in children. Front. Psychol. 6:882. 10.3389/fpsyg.2015.00882 26191012 PMC4488606

[B22] Gantz W. Schwartz N. Angelini J. R. (2007). Television Food Advertising to Children in the United States. Menlo Park, CA: The Kaiser Family Foundation.

[B23] Gearhardt A. N. Yokum S. Harris J. L. Epstein L. H. Lumeng J. C. (2020). Neural response to fast food commercials in adolescents predicts intake. Am. J. Clin. Nutr. 111, 493–502. 10.1093/ajcn/nqz305 31940031 PMC7049532

[B24] Gearhardt A. N. Yokum S. Stice E. Harris J. L. Brownell K. D. (2014). Relation of obesity to neural activation in response to food commercials. Soc. Cogn. Affect. Neurosci. 9, 932–938. 10.1093/scan/nst059 23576811 PMC4090951

[B25] Goris J. M. Petersen S. Stamatakis E. Veerman J. L. (2010). Television food advertising and the prevalence of childhood overweight and obesity: a multicountry comparison. Public Health Nutr. 13, 1003–1012. 10.1017/S1368980009992850 20018123

[B26] Gorn G. J. Goldberg M. E. (1982). Behavioral evidence of the effects of televised food messages on children. J. Consum. Res. 9, 200–205. 10.1086/208913

[B27] Ha O.-R. Lim S.-L. Bruce A. S. (2020). Neural mechanisms of food decision-making in children. Curr. Nutr. Rep. 9, 236–250. 10.1007/s13668-020-00321-5 32720119

[B28] Ha O. R. Bruce A. S. Pruitt S. W. Cherry J. B. Smith T. R. Burkart D.. (2016). Healthy eating decisions require efficient dietary self-control in children: a mouse-tracking food decision study. Appetite 105, 575–581. 10.1016/j.appet.2016.06.027 27349708

[B29] Ha O. R. Killian H. Bruce J. M. Lim S. L. Bruce A. S. (2018). Food advertising literacy training reduces the importance of taste in children's food decision-making: a pilot study. Front. Psychol. 9:1293. 10.3389/fpsyg.2018.01293 30100889 PMC6072865

[B30] Ha O. R. Lim S. L. Bruce J. M. Bruce A. S. (2019). Unhealthy foods taste better among children with lower self-control. Appetite 139, 84–89. 10.1016/j.appet.2019.04.015 31026492

[B31] Harris J. Haraghey K. Lodolce M. Semenza N. (2018). Teaching children about good health? Halo effects in child-directed advertisements for unhealthy food. Pediatr. Obes. 13, 256–264. 10.1111/ijpo.12257 29076259

[B32] Harris J. L. Bargh J. A. Brownell K. D. (2009). Priming effects of television food advertising on eating behavior. Health Psychol. 28:404. 10.1037/a0014399 19594263 PMC2743554

[B33] Hudders L. Cauberghe V. Panic K. (2016). How advertising literacy training affect children's responses to television commercials versus advergames. Int. J. Advert. 35, 909–931. 10.1080/02650487.2015.1090045

[B34] Keller K. L. Kuilema L. G. Lee N. Yoon J. Mascaro B. Combes A.-L.. (2012). The impact of food branding on children's eating behavior and obesity. Physiol. Behav. 106, 379–386. 10.1016/j.physbeh.2012.03.011 22450261

[B35] Kelly B. Halford J. C. Boyland E. J. Chapman K. Bautista-Castaño I. Berg C.. (2010). Television food advertising to children: a global perspective. Am. J. Public Health 100, 1730–1736. 10.2105/AJPH.2009.179267 20634464 PMC2920955

[B36] King N. A. Burley V. J. Blundell J. E. (1994). Exercise-induced suppression of appetite: effects on food intake and implications for energy balance. Eur. J. Clin. Nutr. 48, 715–724. 7835326

[B37] Lim S. L. Cherry J. B. Davis A. M. Balakrishnan S. N. Ha O. R. Bruce J. M. (2016). The child brain computes and utilizes internalized maternal choices. Nat. Commun. 7:11700 10.1038/ncomms11700 27218420 PMC4890300

[B38] Linn S. Novosat C. L. (2008). Calories for sale: food marketing to children in the twenty-first century. Ann. Am. Acad. Polit. Soc. Sci. 615, 133–155. 10.1177/0002716207308487

[B39] Livingstone S. Helsper E. J. (2006). Does advertising literacy mediate the effects of advertising on children? A critical examination of two linked research literatures in relation to obesity and food choice. J. Commun. 56, 560–584. 10.1111/j.1460-2466.2006.00301.x

[B40] Malmelin N. (2010). What is advertising literacy? Exploring the dimensions of advertising literacy. J. Vis. Liter. 29, 129–142. 10.1080/23796529.2010.11674677

[B41] Masterson T. D. Bermudez M. A. Austen M. Lundquist E. Pearce A. L. Bruce A. S.. (2019a). Food commercials do not affect energy intake in a laboratory meal but do alter brain responses to visual food cues in children. Appetite 132, 154–165. 10.1016/j.appet.2018.10.010 30312738 PMC7061687

[B42] Masterson T. D. Stein W. M. Beidler E. Bermudez M. English L. K. Keller K. L. (2019b). Brain response to food brands correlates with increased intake from branded meals in children: an fMRI study. Brain Imaging Behav. 13, 1035–1048. 10.1007/s11682-018-9919-8 29971684 PMC7061688

[B43] Mela D. J. (2001). Determinants of food choice: relationships with obesity and weight control. Obes. Res. 9, 249S−255S. 10.1038/oby.2001.127 11707550

[B44] Moses L. J. Baldwin D. A. (2005). What can the study of cognitive development reveal about children's ability to appreciate and cope with advertising? J. Public Policy Market. 24, 186–201. 10.1509/jppm.2005.24.2.186

[B45] Nelson M. R. (2018). Research on children and advertising then and now: challenges and opportunities for future research. J. Advert. 47, 301–308. 10.1080/00913367.2018.1552218

[B46] Neumark-Sztainer D. Story M. Perry C. Casey M. A. (1999). Factors influencing food choices of adolescents: findings from focus-group discussions with adolescents. J. Am. Dietet. Assoc. 99, 929–937. 10.1016/S0002-8223(99)00222-9 10450307

[B47] Oates C. Blades M. Gunter B. (2002). Children and television advertising: when do they understand persuasive intent? J. Consum. Behav. 1, 238–245. 10.1002/cb.69

[B48] Ogden J. Roy-Stanley C. (2020). How do children make food choices? Using a think-aloud method to explore the role of internal and external factors on eating behaviour. Appetite 147:104551. 10.1016/j.appet.2019.104551 31821839

[B49] Petersen A. C. Crockett L. (1985). Pubertal timing and grade effects on adjustment. J. Youth Adolesc. 14, 191–206. 10.1007/BF02090318 24301176

[B50] Petersen A. C. Crockett L. Richards M. Boxer A. (1988). A self-report measure of pubertal status: reliability, validity, and initial norms. J. Youth Adolesc. 17, 117–133. 10.1007/BF01537962 24277579

[B51] Pettigrew S. Tarabashkina L. Roberts M. Quester P. Chapman K. Miller C. (2013). The effects of television and Internet food advertising on parents and children. Public Health Nutr. 16, 2205–2212. 10.1017/S1368980013001067 23635396 PMC10271572

[B52] Piernas C. Popkin B. M. (2010). Trends in snacking among US children. Health Affairs 29, 398–404. 10.1377/hlthaff.2009.0666 20194979 PMC2837536

[B53] Raghunathan R. Naylor R. W. Hoyer W. D. (2006). The unhealthy = tasty intuition and its effects on taste inferences, enjoyment, and choice of food products. J. Market. 70, 170–184. 10.1509/jmkg.70.4.170

[B54] Robinson T. N. Borzekowski D. L. Matheson D. M. Kraemer H. C. (2007). Effects of fast food branding on young children's taste preferences. Archiv. Pediatr. Adolesc. Med. 161, 792–797. 10.1001/archpedi.161.8.792 17679662

[B55] Rozendaal E. Buijs L. Reijmersdal E. A. V. (2016). Strengthening children's advertising defenses: the effects of forewarning of commercial and manipulative intent. Front. Psychol. 7:1186. 10.3389/fpsyg.2016.01186 27551271 PMC4976102

[B56] Rozendaal E. Buijzen M. Valkenburg P. (2010). Comparing children's and adults' cognitive advertising competences in the Netherlands. J. Child. Media 4, 77–89. 10.1080/17482790903407333

[B57] Rozendaal E. Buijzen M. Valkenburg P. M. (2012). Think-aloud process superior to thought-listing in increasing children's critical processing of advertising. Hum. Commun. Res. 38, 199–221. 10.1111/j.1468-2958.2011.01425.x

[B58] Rozendaal E. Lapierre M. A. Van Reijmersdal E. A. Buijzen M. (2011). Reconsidering advertising literacy as a defense against advertising effects. Media Psychol. 14, 333–354. 10.1080/15213269.2011.620540

[B59] Russell S. J. Croker H. Viner R. M. (2019). The effect of screen advertising on children's dietary intake: a systematic review and meta-analysis. Obes. Rev. 20, 554–568. 10.1111/obr.12812 30576057 PMC6446725

[B60] Shannon C. Story M. Fulkerson J. A. French S. A. (2002). Factors in the school cafeteria influencing food choices by high school students. J. School Health 72, 229–234. 10.1111/j.1746-1561.2002.tb07335.x 12212407

[B61] Smith R. Kelly B. Yeatman H. Boyland E. (2019). Food marketing influences children's attitudes, preferences and consumption: a systematic critical review. Nutrients 11:875 10.3390/nu11040875 PMC6520952 31003489

[B62] Spielvogel I. Matthes J. Naderer B. Karsay K. (2018). A treat for the eyes. An eye-tracking study on children's attention to unhealthy and healthy food cues in media content. Appetite 125, 63–71. 10.1016/j.appet.2018.01.033 29410047

[B63] Stice E. Yokum S. (2016). Gain in body fat is associated with increased striatal response to palatable food cues, whereas body fat stability is associated with decreased striatal response. J. Neurosci. 36, 6949–6956. 10.1523/JNEUROSCI.4365-15.2016 27358453 PMC4926241

[B64] Utter J. Scragg R. Schaaf D. (2006). Associations between television viewing and consumption of commonly advertised foods among New Zealand children and young adolescents. Public Health Nutr. 9, 606–612. 10.1079/PHN2005899 16923292

[B65] Vandevijvere S. Soupen A. Swinburn B. (2017). Unhealthy food advertising directed to children on New Zealand television: extent, nature, impact and policy implications. Public Health Nutr. 20, 3029–3040. 10.1017/S1368980017000775 28545596 PMC10261618

[B66] Whalen R. Harrold J. Child S. Halford J. Boyland E. (2019). Children's exposure to food advertising: the impact of statutory restrictions. Health Promot. Int. 34, 227–235. 10.1093/heapro/dax044 29092014

