# Supplementary material for: Promoting Resilience to Food Commercials Decreases Susceptibility to Unhealthy Food Decision-Making
Source: Front Psychol. 2020 Dec 2;11:599663. doi: 10.3389/fpsyg.2020.599663 (PMC7738621; doi:10.3389/fpsyg.2020.599663)
Supplement: Supplementary file 1 [file Data_Sheet_1.docx]

Table S1

*Descriptive Statistics of Food Commercial Questionnaires*

|  |  |  |  | Group | | | | |  |
| --- | --- | --- | --- | --- | --- | --- | --- | --- | --- |
|  | Component | Sessions |  | Intervention | |  | Control | |  |
|  |  |  |  |  |  |  |  |  |  |
|  |  |  |  | M | (SD) |  | M | (SD) |  |
|  |  |  |  |  |  |  |  |  |  |
| Attitude Toward Commercials |  | 1 |  | 3.08 | (.90) |  | 3.18 | (.72) |  |
|  | Belief | 2 |  | 2.67 | (.95) |  | 2.84 | (1.06) |  |
|  |  | 3 |  | 2.85 | (.84) |  | 2.89 | (.85) |  |
|  |  | 4 |  | 2.75 | (.78) |  | 3.02 | (.98) |  |
|  |  |  |  |  | | | | |  |
|  | Liking | 1 |  | 2.80 | (.44) |  | 2.71 | (.55) |  |
|  |  | 2 |  | 2.57 | (.63) |  | 2.47 | (.60) |  |
|  |  | 3 |  | 2.66 | (.64) |  | 2.51 | (.66) |  |
|  |  | 4 |  | 2.61 | (.78) |  | 2.43 | (.64) |  |
|  |  |  |  |  | | | | |  |
|  | Positive Attitude | 1 |  | 4.83 | (.95) |  | 3.06 | (.89) |  |
|  |  | 2 |  | 4.33 | (.79) |  | 2.90 | (1.02) |  |
|  |  | 3 |  | 4.33 | (.74) |  | 2.93 | (.92) |  |
|  |  | 4 |  | 4.17 | (.91) |  | 3.09 | (.79) |  |
|  |  |  |  |  | | | | |  |
| Commercial Impact | Food Preferences | 1 |  | 3.78 | (1.06) |  | 2.94 | (1.31) |  |
|  |  | 2 |  | 2.65 | (1.22) |  | 2.83 | (1.38) |  |
|  |  | 3 |  | 2.94 | (1.30) |  | 2.61 | (1.24) |  |
|  |  | 4 |  | 3.06 | (1.43) |  | 2.39 | (1.24) |  |
|  |  |  |  |  | | | | |  |
|  | Food Choices | 1 |  | 3.22 | (1.31) |  | 3.22 | (1.11) |  |
|  |  | 2 |  | 3.41 | (1.18) |  | 2.78 | (1.31) |  |
|  |  | 3 |  | 3.06 | (1.25) |  | 3.00 | (1.50) |  |
|  |  | 4 |  | 3.17 | (1.30) |  | 3.00 | (1.65) |  |

Table S2

*Descriptive Statistics of Think-Aloud Responses*

|  |  |  |  | Responses Toward Commercials | | | | |  | Responses Toward Narratives | | | | |
| --- | --- | --- | --- | --- | --- | --- | --- | --- | --- | --- | --- | --- | --- | --- |
|  |  |  |  | Group | | | | |  | Group | | | | |
|  | Component | Sessions |  | Intervention | |  | Control | |  | Intervention | |  | Control | |
|  |  |  |  |  |  |  |  |  |  |  |  |  |  |  |
|  |  |  |  | M | (SD) |  | M | (SD) |  | M | (SD) |  | M | (SD) |
|  |  |  |  |  |  |  |  |  |  |  |  |  |  |  |
| Number of Reponses |  | 1 |  | 9.6 | (6.5) |  | 8.3 | (8.5) |  | 5.2 | (4.9) |  | 2.1 | (2.6) |
|  |  | 2 |  | 11.2 | (5.5) |  | 10.2 | (6.3) |  | 6.3 | (4.7) |  | 2.7 | (3.7) |
|  |  | 3 |  | 11.3 | (7.2) |  | 9.3 | (4.5) |  | 6.7 | (4.7) |  | 2.7 | (2.4) |
|  |  | 4 |  | 9.1 | (6.3) |  | 7.2 | (5.8) |  | 6.3 | (5.9) |  | 2.5 | (3.5) |
|  |  |  |  |  | | | | |  |  | | | | |
| Number of Relevant, Recipient-Generated Responses | Cognitive | 1 |  | 1.7 | (2.1) |  | 1.1 | (2.0) |  | 2.3 | (3.0) |  | 0.6 | (1.2) |
|  |  | 2 |  | 2.5 | (2.4) |  | 1.4 | (1.3) |  | 3.2 | (3.2) |  | 1.0 | (1.4) |
|  |  | 3 |  | 1.4 | (1.8) |  | 1.8 | (1.8) |  | 3.4 | (3.1) |  | 0.5 | (0.9) |
|  |  | 4 |  | 1.4 | (1.5) |  | 1.3 | (1.8) |  | 2.7 | (3.4) |  | 0.8 | (1.9) |
|  |  |  |  |  | | | | |  |  | | | | |
|  | Affective | 1 |  | 4.8 | (4.2) |  | 4.9 | (6.3) |  | 1.5 | (1.5) |  | 1.2 | (1.5) |
|  |  | 2 |  | 5.5 | (4.4) |  | 6.9 | (5.2) |  | 2.1 | (2.0) |  | 1.2 | (2.0) |
|  |  | 3 |  | 5.3 | (4.2) |  | 5.4 | (3.9) |  | 2.2 | (1.7) |  | 1.6 | (2.2) |
|  |  | 4 |  | 3.9 | (2.9) |  | 3.9 | (3.9) |  | 1.9 | (2.3) |  | 0.9 | (1.9) |
|  |  |  |  |  | | | | |  |  | | | | |
| Ratio of Relevant, Recipient-Generated Responses (%) | Negative Cognitive | 1 |  | 11.1 | (32.3) |  | 22.2 | (42.8) |  | 28.3 | (43.0) |  | 11.1 | (32.3) |
|  |  | 2 |  | 48.2 | (47.1) |  | 38.9 | (50.2) |  | 38.7 | (42.6) |  | 23.5 | (43.7) |
|  |  | 3 |  | 27.8 | (46.1) |  | 42.2 | (47.9) |  | 42.4 | (43.8) |  | 11.8 | (33.2) |
|  |  | 4 |  | 43.5 | (48.2) |  | 37.0 | (48.4) |  | 52.4 | (46.9) |  | 25.0 | (42.9) |
|  |  |  |  |  | | | | |  |  | | | | |
|  | Negative Affective | 1 |  | 43.0 | (33.5) |  | 38.0 | (41.3) |  | 29.2 | (38.6) |  | 20.0 | (39.4) |
|  |  | 2 |  | 42.8 | (37.3) |  | 46.7 | (39.4) |  | 29.5 | (41.5) |  | 30.4 | (44.2) |
|  |  | 3 |  | 42.6 | (38.5) |  | 53.9 | (41.5) |  | 20.4 | (36.0) |  | 18.4 | (35.9) |
|  |  | 4 |  | 50.1 | (35.7) |  | 41.8 | (39.6) |  | 44.3 | (41.9) |  | 22.2 | (39.2) |
